# A Case of Rathke's Cleft Cyst Associated with Transient Central Adrenal Insufficiency and Masked Diabetes Insipidus

**DOI:** 10.1155/2014/693294

**Published:** 2014-11-06

**Authors:** Masahiro Asakawa, Rina Chin, Yoshihiro Niitsu, Tetsuo Sekine, Arisa Niwa, Atsuko Miyake, Naoko Inoshita, Mitsunobu Kawamura, Yoshihiro Ogawa, Yukio Hirata

**Affiliations:** ^1^Department of Endocrinology and Metabolism, Tokyo Teishin Hospital, 2-14-23, Fujimi, Chiyoda-ku, Tokyo 102-0071, Japan; ^2^Department of Endocrinology and Metabolism, Tokyo Kyosai Hospital, 2-3-8, Nakameguro, Meguro-ku, Tokyo 153-0061, Japan; ^3^Department of Pathology, Toranomon Hospital, 2-2-2, Toranomon, Minato-ku, Tokyo 105-0001, Japan; ^4^Department of Molecular Endocrinology and Metabolism, Tokyo Medical and Dental University Hospital, 1-5-45, Yushima, Bunkyo-ku, Tokyo 113-8510, Japan; ^5^Institute of Biochemical Research and Innovation Hospital, 2-2 Minatoshima-Minamicho, Chuo-ku, Kobe, Hyogo 650-0047, Japan

## Abstract

A 73-year-old woman admitted to our hospital because of headache, poor appetite, malaise, weight loss, and vomiting was found to have central adrenal insufficiency and thyrotoxicosis due to silent thyroiditis. Polyuria developed after replacement with glucocorticoid (masked diabetes insipidus), which was controlled with nasal administration of desmopressin. Magnetic resonance imaging of the brain showed a large cystic pituitary mass (18 × 18 × 12 mm) extending suprasellarly to the optic chiasm. Transsphenoidal surgery revealed that the pituitary tumor was Rathke's cleft cyst. Following surgery, replacement with neither glucocorticoid nor desmopressin was needed any more. Therefore, it is suggested that Rathke's cleft cyst is responsible for the masked diabetes insipidus and the central insufficiency. Furthermore, it is speculated that thyrotoxicosis with painless thyroiditis might induce changes from subclinical adrenal insufficiency to transiently overt insufficiency.

## 1. Introduction

Rathke's cleft cyst (RCC) is defined as cystic sellar and suprasellar lesions derived from remnants of Rathke's pouch which is lined with cuboidal or columnar epithelium. Normally, Rathke's pouch is closed off by cell proliferation of the anterior and the posterior lobes of the pituitary gland during fetal development, leaving a thin residual cleft in the gland. A failure of pouch obliteration results in cysts or cystic remnants at the interface between the anterior and posterior pituitary lobe [1, 2]. RCC, found frequently (13–22%) in normal pituitary glands at autopsy [2], is sometimes identified incidentally by magnetic resonance imaging (MRI) of the head [3]. RCC, although usually asymptomatic, may accompany various symptoms when it enlarges to compress the optic chiasm, hypothalamus, and pituitary gland [3–5]. When inflammation within the cysts involves the adjacent pituitary gland, it may cause symptoms even without mechanical compression [4–6]. The most common symptoms include headache, visual disturbance, and hypopituitarism, and, less commonly, diabetes insipidus [2, 5, 7, 8]. Such symptoms may resolve or be improved by transsphenoidal surgery with complete cyst evacuation and partial wall excision [3, 5–9]. Herein, we report a case of RCC associated with transient central adrenal insufficiency and masked diabetes insipidus possibly induced by silent thyroiditis, both of which improved after surgical removal of RCC.

## 2. Case Presentation

A 73-year-old woman was admitted to our hospital because of severe headache, anorexia, malaise, weight loss, and vomiting. She had progressive headache and lost weight by 6 kg during about a half year. One month prior to admission, she began to have frequent vomiting. Her past history was noncontributory, and her family history showed hepatocellular carcinoma in mother and stomach cancer in siblings. She did not smoke and drank little alcohol.

She was 160 cm tall and weighed 59.5 kg, body temperature 36.8°C. Her blood pressure was 108/65 mmHg with a regular sinus rhythm of 115 bpm. Physical examinations of the chest, abdomen, and extremities were normal. Neither axillar or pubic hair loss nor abnormal skin pigmentation was noted. Ophthalmologic examination revealed left temporal hemianopia and optic nerve atrophy and right incomplete hemianopia.

Laboratory data showed slightly high serum sodium (149 mEq/L) and high plasma osmolality (296 mOsm/L). Liver and renal functions were normal. Endocrine data (Table 1) showed very low levels of both ACTH and cortisol consistent with secondary adrenal insufficiency. The suppressed TSH level in the presence of elevated free T3 and free T4 levels is consistent with primary hyperthyroidism. Other pituitary hormones included low GH level and normal IGF-1 level for her age, elevated PRL level, low gonadotropin levels, and relatively low arginine vasopressin (AVP) level for high plasma osmolality. Anti-thyroid peroxidase antibody, TSH receptor antibody, and thyroid-stimulating antibody were all negative except positive anti-thyroglobulin antibody. ^123^I-Thyroid scintigraphy showed a markedly reduced uptake rate (0.24%). These thyroid function data are compatible with the diagnosis of thyrotoxicosis due to silent thyroiditis.

**Figure 1:**
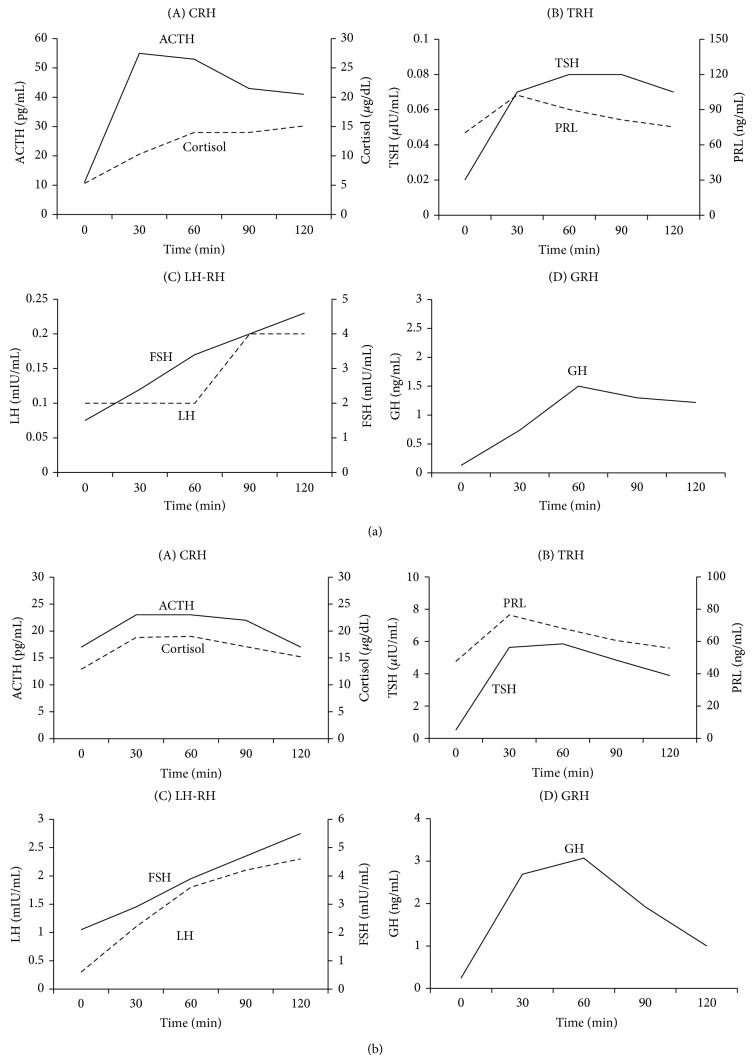
Dynamic endocrine tests before (a) and after (b) surgery. Stimulation with (A) CRH (100 *μ*g), (B) TRH (500 *μ*g), (C) LH-RH (100 *μ*g), and (D) GRH (100 *μ*g) is shown.

**Figure 2:**
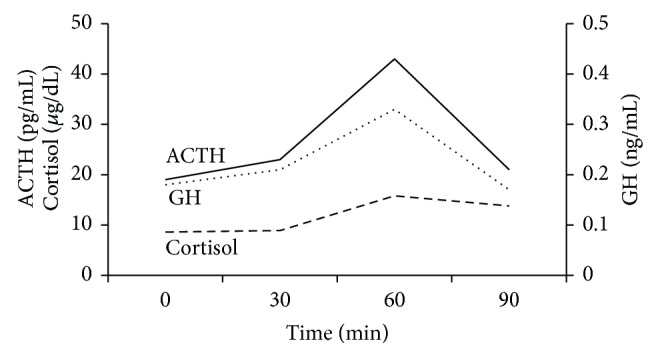
Insulin tolerance test. Regular insulin (0.1 U/Kg) was intravenously administered as a bolus with a nadir plasma glucose level (28 mg/dL) at 30 minutes.

The dynamic endocrine stimulation tests with CRH, LH-RH, and GRH performed just after admission except TRH test which was performed after improvement of thyrotoxicosis are shown in Figure 1(a): a normal response of ACTH and delayed response of cortisol after CRH, low responses of TSH and PRL after TRH, a low response of LH and a delayed response of FSH after LH-RH, and a low response of GH after GRH. Insulin tolerance test (0.1 U/kg) showed normal responses of both ACTH and cortisol and a low response of GH (Figure 2). Rapid ACTH test (Table 1) showed a low response of cortisol consistent with secondary adrenal insufficiency. These data are compatible with the diagnosis of hypopituitarism due to the impairment at pituitary and/or hypothalamic level.

**Figure 3:**
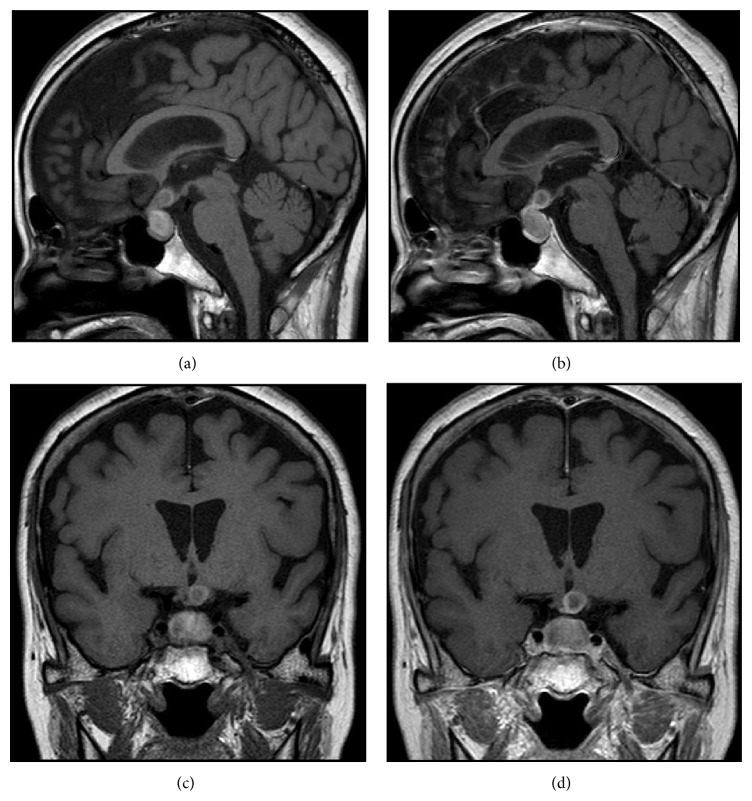
Brain MRI (T1-weighted images). Sagittal views of (a) plain and (b) Gd-enhanced images and coronal views of (c) plain and (d) Gd-enhanced images are shown.

MRI of the brain revealed a large cystic pituitary mass (18 × 12 × 12 mm) extending suprasellarly to the optic chiasm (Figure 3); high intensity signal on T1 weighted-images and the rim effect enhanced by gadolinium contrast are suggestive of RCC.

Based on the diagnosis of central adrenal insufficiency, she was treated with glucocorticoid replacement (hydrocortisone: 10 mg), which markedly increased urine volume (5000–6000 mL/day) associated with high serum osmolality (301 mOsm/L), low urine osmolality (91 mOsm/L), and low AVP level (0.95 pg/mL). Thus, development of overt diabetes insipidus following glucocorticoid replacement is compatible with the diagnosis of “masked” diabetes insipidus. Nasal administration of DDAVP (15 *μ*g) dramatically decreased urine volume (1500–2000 mL/day). Furthermore, because her thyrotoxicosis became hypothyroidism (TSH: 2.13 *μ*IU/mL, free T3: 1.1 pg/mL, free T4: 0.47 ng/dL) during the natural course, she was treated with thyroid hormone (levothyroxine: 25 *μ*g). After replacement with levothyroxine, most of her nonspecific symptoms, such as poor appetite, malaise, weight loss, and vomiting, gradually improved, but her headache persisted.

She underwent transsphenoidal surgery with complete cyst evacuation and partial wall excision; RCC was confirmed histopathologically. Postoperatively, her headache resolved, but bitemporal hemianopia persisted. Postoperative endocrine data (Table 1) showed elevated levels of ACTH, cortisol, TSH, and AVP but low levels of thyroid hormones and gonadotropin and normal PRL level. Postoperative dynamic endocrine stimulation tests (Figure 1(b)) showed normal responses of ACTH and cortisol after CRH, a delayed response of TSH and a low response of PRL after TRH, delayed responses of LH and FSH after LH-RH, and a normal response of GH after GRH. Postoperative rapid ACTH test (Table 1) showed a normal response of cortisol after ACTH stimulation. Based on the postoperative endocrine data, replacements with glucocorticoid and desmopressin were stopped, while thyroid hormone was continued.

## 3. Discussion

RCC, a benign cystic pituitary mass, is often detected incidentally by MRI of the brain. RCC is usually asymptomatic, but when it enlarges and/or is associated with inflammation, various symptoms, such as headache, visual disturbance, and endocrine dysfunction, may occur. Endocrine dysfunction is observed in 40–60% of patients with a symptomatic RCC [9–12], among which hypopituitarism is most common (75–100%), but diabetes insipidus is less common (0–20%) [3, 5, 8]. A key mechanism for pituitary dysfunction caused by a large cystic RCC has been considered to be due to its compression to normal pituitary gland [13], although intrasellar RCC seems unlikely to cause such mechanical compression. Alternatively, RCC-associated inflammatory change in the pituitary gland may have caused pituitary dysfunction [5]. In fact, such inflammatory change has been assumed to result from the leakage of its cyst contents into the pituitary gland [5].

The major symptoms and signs of headache and visual disturbance in this case are due to the suprasellar pituitary tumor compressing optic chiasm as demonstrated by brain MRI. The concomitant clinical manifestations of anorexia, malaise, and weight loss suggest the possible hypopituitarism by the pituitary tumor as evidenced by the endocrine data with low basal levels of pituitary hormones (ACTH, TSH, GH, LH, and FSH) and hyperprolactinemia. The dynamic endocrine data with low and/or delayed responses of GH by GRH, LH and FSH by LH-RH, and TSH by TRH suggest the pituitary and/or the hypothalamic lesions. On the other hand, normal responses of ACTH and cortisol by CRH stimulation and insulin tolerance test and a low response of cortisol after ACTH stimulation are consistent with hypothalamic rather than pituitary lesion. Thus, hypopituitarism in this case is most likely caused by the impairment by pituitary and/or hypothalamic lesions. Her nonspecific symptoms (anorexia, malaise, and weight loss) are due to either adrenal insufficiency or thyrotoxicosis due to silent thyroiditis. Since thyrotoxicosis accelerates the metabolism of cortisol to induce adrenal insufficiency, it is possible to speculate that subclinical adrenal insufficiency was actualized in overt insufficiency, triggered by thyrotoxicosis in our case.

Soon after replacement with glucocorticoid, she had markedly increased urine volume with elevated serum osmolality, low urine osmolality, and decreased vasopressin release, suggesting the onset of overt diabetes insipidus. Masked diabetes insipidus is defined as the condition in which polyuria due to diabetes insipidus is masked by glucocorticoid deficiency. The underlying mechanisms of masked diabetes insipidus are accounted by various effects of glucocorticoid, such as inhibition of vasopressin release [14], reduced water absorption at the proximal tubule [15], and stimulation of atrial natriuretic peptide secretion to enhance the diuretic effect [16]. In the present case, vasopressin release impaired by the compression and/or inflammation of the pituitary stalk by RCC should have caused overt diabetes insipidus, which could have been masked by the transient glucocorticoid deficiency due to central adrenal insufficiency.

The cyst wall of RCC is histopathologically characterized by lining by cuboidal or columnar epithelial cells. However, the presence of a small amount of squamous epithelium in this case suggests that squamous metaplasia occurs due to inflammation of the RCC. In fact, it has been reported that squamous epithelium is identified in 45% with symptomatic RCC [9]. Furthermore, the presence of inflammatory cells in the cyst wall in this case was suspected by the high-intensity mass on the T1-weighted images, reflecting mucous materials within the cyst, often associated with chronic inflammation, which may potentially cause irreversible endocrine dysfunction [17]. Therefore, it is suggested that the pituitary dysfunction was caused not only by compression by the mass, but also by inflammation.

After surgery, pituitary endocrine dysfunctions improved promptly. In particular, replacement with glucocorticoid became unnecessary on postoperative 5th day and then that with desmopressin became unnecessary on postoperative 60th day, suggesting reversible changes of compression and/or inflammation by RCC. In contrast, her headache resolved after surgery without any improvement of visual disturbance (temporal hemianopia). It has been reported that, during the clinical course of RCC, headache, visual disturbance, and pituitary dysfunction improved after surgery in 80–95%, 65–98%, and 15–50% cases, respectively [3, 8, 9, 12, 18]. Our case required thyroid hormone replacement due to primary hypothyroidism resulting from silent thyroiditis. Although hypothyroidism following silent thyroiditis in most cases improves within three months, hypothyroidism remains with requirement of permanent thyroid hormone replacement in 10–15% cases.

In summary, we report a case of RCC associated with transient central adrenal insufficiency and masked diabetes insipidus with concomitant silent thyroiditis. Although RCC is usually asymptomatic and needs no treatment, surgical treatment should be considered if persistent symptoms such as headache, visual disturbance, or endocrine dysfunction are present. In our case, RCC with endocrine dysfunction was treated successfully by transsphenoidal surgery, so that replacement with glucocorticoid and desmopressin is no longer needed. She is free from recurrence so far, but careful follow-up is necessary because the presence of squamous metaplasia has been reported to be a predictor of its recurrence [12].

## Figures and Tables

**Table 1:** Preoperative and postoperative basal endocrine data.

Basal hormone level	Before surgery	Postoperative day 5	Postoperative day 60
ACTH (pg/mL)	5	11.9	17
Cortisol (*μ*g/dL)	<1.0	25.2	12.9
TSH (*μ*IU/mL)	0.01	0.66	1.1
Free T3 (pg/mL)	6.6	1.5	1.5
Free T4 (ng/dL)	1.82	0.42	0.34
GH (ng/mL)	0.18	0.6	0.24
IGF-1 (ng/mL)	67.8	39.0	87.6
PRL (ng/mL)	116.7	35.1	47.6
LH (mIU/mL)	0.1	0.3	0.1
FSH (mIU/mL)	2.5	2.5	1.5
Renin activity (ng/mL/hr)	6.7	—	2.3
Aldosterone (pg/mL)	151	—	126
DHEA-S (*μ*g/dL)	13	—	—
Estradiol (pg/mL)	16	—	—
AVP (pg/mL)	1.19	—	2.4

ACTH stimulation test		—	
Cortisol (*μ*g/dL) 0 (min)	1.0	—	12.1
Cortisol (*μ*g/dL) 30 (min)	7.5	—	23.3
Cortisol (*μ*g/dL) 60 (min)	10.2	—	28.5

ACTH: adrenocorticotrophic hormone; TSH: thyroid stimulating hormone; T3: triiodothyronine; T4: thyroxine; GH: growth hormone; IGF-1: insulin-like growth factor-1; LH: luteinizing hormone; FSH: follicle-stimulating hormone; DHEA-S: dehydroepiandrosterone-sulfate; AVP: arginine vasopressin.
